# Within‐Host Environmental Heterogeneity Is Associated With Phenotypic but Not Genomic Diversity in *Wolbachia* Endosymbionts

**DOI:** 10.1111/1758-2229.70286

**Published:** 2026-02-16

**Authors:** Romain Pigeault, Yann Dussert, Raphaël Jorge, Theo Ulve, Marie Panza, Maryline Raimond, Carine Delaunay, Willy Aucher, Thierry Berges, David Ogereau, Bouziane Moumen, Jean Peccoud, Richard Cordaux

**Affiliations:** ^1^ Université de Poitiers, CNRS, EBI, UMR 7267 Poitiers France; ^2^ Génomique Métabolique, Genoscope, Institut François Jacob, CEA, CNRS, Univ Evry, Université Paris‐Saclay Evry France; ^3^ Université Lyon 1, CNRS, VetAgroSup, Laboratoire de Biométrie et Biologie Evolutive, UMR 5558 Villeurbanne France; ^4^ INSA Lyon, INRAE, Biologie Fonctionnelle, Insectes et Interactions, UMR203 Villeurbanne France; ^5^ Université Paris‐Saclay, CNRS, IRD, UMR Évolution Génomes Comportement Écologie Gif‐sur‐Yvette France

**Keywords:** acclimation, adaptation, feminising *Wolbachia*, gene conversion, infection dynamics, structural variation, subpopulation, tissue tropism, variant calling, within‐host evolution

## Abstract

Hosts represent complex environments where different tissues may act as distinct ecological niches, imposing different constraints that may shape parasite ecology and evolution. Such within‐host heterogeneity can generate phenotypic diversity with consequences for virulence and transmission. Our aim was to determine whether the constraints associated with infecting different host tissues lead to the coexistence of multiple parasite sub‐populations with distinct phenotypes. We tested this hypothesis using the widespread bacterial endosymbiont *Wolbachia*. We injected bacteria isolated from three tissues of the common pill‐bug into uninfected individuals and tracked temporal changes in *Wolbachia* load in the recipient host tissues, as well as the virulence associated with each bacterial source. Our results show that colonisation success depends on the tissue of origin of the injected *Wolbachia*. Genome resequencing did not detect any genetic variation associated with variation in bacterial replication rate, which thus likely results from phenotypic plasticity. Indeed, no recurrent tissue‐specific variants were detected, and our conservative filtering pipeline retained only one substitution and one gene conversion event. These findings highlight the genomic stability of *Wolbachia* across host environments while demonstrating that within‐host diversification can occur without genetic divergence. More broadly, they underscore how microenvironmental variation within hosts can shape parasite ecology.

## Graphical abstract

(A) Wolbachia's success in colonising new hosts depends on the tissue they originate from. (B) Genome resequencing revealed that differences in colonisation success were not due to genetic change but instead to phenotypic plasticity. (C) Six months after injection, *Wolbachia*'s tissue origin no longer influenced their ability to maintain infection.

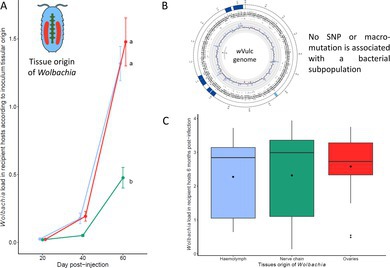

## 1 Introduction

Among the many factors that influence species richness and community structure, environmental heterogeneity is generally considered to be a particularly important driver (Ben‐Hur and Kadmon 2020; Stein et al. 2014; Stein and Kreft 2015). Since heterogeneous environments offer a variety of ecological niches and refuges from adverse conditions, species richness and persistence should increase with greater spatial heterogeneity (Ben‐Hur and Kadmon 2020; Burnett et al. 1998; Stein et al. 2014; Yang et al. 2015). Environmental heterogeneity not only favours phenotypic plasticity (Edelaar et al. 2017; Lázaro‐Nogal et al. 2015) but it also promotes genetic diversity within species through diversifying selection (Houle et al. 2020; Huang et al. 2017; Mcdonald and Ayala 1974; Rainey and Travisano 1998), which may underpin local adaptations (Huang et al. 2016; Qian et al. 2024; Trevail et al. 2021; van Houte et al. 2021). However, most studies that investigated relationships between environmental heterogeneity and diversity have focused on free‐living species (e.g., Langenheder and Lindström 2019; Stein and Kreft 2015). The relationship between environmental heterogeneity and both endoparasite species diversity and phenotypic variability remains underexplored.

For endoparasites, environmental heterogeneity can exist both within individual hosts and across different hosts. Variation among individuals within host populations is known to shape parasite evolution (Johnson et al. 2016; Regoes et al. 2000; White et al. 2020), driving rapid diversification (Johnson et al. 2016; Kamiya et al. 2014). However, a single host individual is treated by most theoretical and empirical studies on endoparasite evolution as a homogeneous environment—a single population of target cells without any structure—neglecting heterogeneities in cell type, immune response, nutrient supply or microbiota composition. Yet, several parasites exploit a heterogeneous environment composed of different biological tissues and fluids which form a spatially structured fitness landscape for the parasite. Successful colonisation of different microenvironments, with their contrasted constraints, may involve the expression or selection of different parasitic phenotypes leading to within‐host differentiation into several sub‐populations (Didelot et al. 2016). This phenotypic variability can reflect phenotypic plasticity (e.g., expression plasticity, Wernegreen and Wheeler 2009), selection of genetic variants initially present in the inoculum (Chrostek and Teixeira 2018) or of new variants arising from spontaneous mutations (Ailloud et al. 2019).

The co‐occurrence of polymorphic sub‐populations of parasites in different tissues has been described in several biological models. Gastric biopsies from multiple stomach regions of *Helicobacter pylori* ‐infected hosts showed location‐specific evolution of the bacteria, suggesting the existence of structured niches with distinct selective pressures within the stomach (Ailloud et al. 2019). In other cases, such as *Pseudomonas aeruginosa* or *Mycobacterium tuberculosis* chronic infections, the emergence of polymorphic sub‐populations seems to be driven by tissue‐specific adaptation (Jorth et al. 2015; Lieberman et al. 2016). Some within‐host parasite sub‐populations may constitute a reservoir from which new virulent variants can emerge (Bessière and Volmer 2021), and which can delay clearance of parasite infection by the host immune system or drugs (Avettand‐Fenoel et al. 2011; Clement et al. 2005; Obaldia et al. 2018). Understanding the evolutionary forces and mechanisms at the origin of within‐host parasite diversity is essential, yet it is rarely studied. Descriptive studies (e.g., long‐term ambulatory monitoring of chronically infected patients) cannot trace the divergence of variants found within a host and cannot identify the drivers of their diversification without controlling or assessing the composition of the infectious inoculum and ensuring the absence of subsequent infections. For this purpose, vertically transmitted parasites are particularly useful, as their transmission mode ensures the common origin of all within‐host parasite sub‐populations.

Among the most widespread vertically transmitted endosymbionts in animals is *Wolbachia pipientis* . This primarily maternally transmitted intracellular alpha‐proteobacterium is widely distributed among arthropods and has been extensively studied with respect to its peculiar effects on its hosts (Kaur et al. 2021; LePage and Bordenstein 2013; Porter and Sullivan 2023). *Wolbachia* manipulates host reproduction in various ways, including the feminization of chromosomal male embryos, parthenogenesis, male killing and sperm–egg incompatibility (Werren et al. 2008). This endosymbiont can also have positive or negative effects on other aspects of the host's life cycle (e.g., immunity, senescence) and influence the evolution of the host genome through horizontal gene transfer (Cordaux and Gilbert 2017; Depeux et al. 2024; Sicard et al. 2014). The diversity of effects of *Wolbachia* on its hosts certainly relates to its wide distribution within infected individuals (Sicard et al. 2014). In addition to colonising the female germline, ensuring vertical transmission, *Wolbachia* has indeed been observed in all major tissues (e.g., nerve chain, immune and digestive compartments, Fischer et al. 2011; Dittmer et al. 2014; Pietri et al. 2016) of many arthropod species. The presence of vertically transmitted endosymbionts in somatic tissues may seem paradoxical, especially considering that systemic infection can decrease host fitness. Given this disadvantage (i.e., fitness alignment), these extensive somatic localizations must somehow benefit the parasite. Localization of *Wolbachia* in somatic tissues could influence host biology in such a way that favours its vertical and, possibly, horizontal transmission. However, the ability of the bacteria to colonise and establish in different tissular microenvironments could involve the expression or selection of different phenotypes.

To investigate this hypothesis, we assessed the relationship between tissue environment heterogeneity and phenotype variability in the *wVulC* feminising *Wolbachia* strain, which infects the common pill‐bug *Armadillidium vulgare* (Crustacea, Isopoda, Armadillidiidae, Cordaux et al. 2004). This unconventional symbiotic association constitutes a unique model to study the impact of within‐host environmental heterogeneity on parasite evolution, as vertical transmission ensures the common origin of all within‐host *Wolbachia* subpopulations and the size and relatively long lifespan of terrestrial isopods (2–3 years) facilitate the spatiotemporal monitoring of *Wolbachia* evolution within host tissues. We focused on three *Wolbachia* subpopulations that naturally colonise three different tissues. These tissues were selected for their physiological characteristics and the benefits that *Wolbachia* could gain by colonising them: (i) gonads, which are a prime target for vertical transmission, (ii) haemocytes, as these circulating immune cells could contribute to spread *Wolbachia* to different host tissues (Braquart‐Varnier et al. 2015) and, in very rare cases, enable/facilitate its horizontal transmission (Rigaud and Juchault 1995) and (iii) the nerve chain, as the localization of *Wolbachia* in the brain and nerve chord could induce adaptive modifications of host behaviour (Bi and Wang 2020; Templé and Richard 2015). We hypothesized that the main phenotypic variation between bacteria sub‐populations colonising these tissues pertained to their replication rate. A high replication rate in vital tissues, such as the nerve chain, could disrupt tissue structure and reduce host lifespan (Le Clec'h et al. 2012; Strunov and Kiseleva 2016). Thus, we expected *Wolbachia* populations from the host nerve system to show lower replication rates than those from the other tissues. We tested this hypothesis by injecting *Wolbachia* extracted from the three tissue types into uninfected *A. vulgare* individuals, and by monitoring their growth rates in their new hosts. Indeed, adult terrestrial isopods are well suited for transfection experiments: their large size makes them easy to handle, and the injection puncture heals easily. We then tested whether *Wolbachia* sub‐populations were adapted and not merely acclimated to their tissue types. In this case, we expected to find recurrent tissue‐specific genomic variation among infected individuals. We tested this prediction by sequencing the whole genomes of focal bacterial sub‐populations.

## 2 Experimental Procedures

### 2.1 Biological Models

All *A. vulgare* individuals used in this experiment were reared as previously described (Sicard et al. 2010) and did not reproduce during the study. Given that the *Wolbachia* strain used in this study is feminising, all the individuals used were females. As *Wolbachia* is not cultivable, phenotyping sub‐populations requires trans‐infection of an uninfected host with bacterial isolates from naturally infected animals. For this purpose, we used a common pill‐bug source line (WXw) infected by the *w*VulC *Wolbachia* strain (source individuals) and a naturally uninfected recipient line (WXa, recipient individuals) into which we have injected bacteria. The two laboratory lines came from the same original population as *A. vulgare* sampled in Helsingør (Denmark).

### 2.2 Experimental Infection

To characterise the phenotype (i.e., virulence and change in *Wolbachia* load through time) of the different *Wolbachia* sub‐populations associated to three different tissues of their native host, five batches of trans‐infection were carried out. Three batches (i.e., three biological replicates) were used to monitor early infection dynamics within the recipient host (Days 0–60 post‐infection, trans‐infection experiment 1), and two batches were used to estimate bacterial persistence in tissues (i.e., two biological replicates, Day 200 post‐infection, trans‐infection experiment 2). For each batch, one tissue suspension was prepared from each of the following tissues: haemolymph, ovaries and nerve chain. Haemolymph (15–20 μL/individual) and tissues were collected from five source females from the WXw line (infection status previously validated by quantitative PCR, see Section 2.4). To prevent contamination of the nerve chain and ovaries by haemolymph, in which these tissues are immersed, the tissues were rinsed in fresh Ringer's solution (1.4 mM CaCl_2_, 2.4 mM HNaCO_3_, 2 mM KCl, 0.4 M NaCl) immediately after dissection. For each suspension, tissues were crushed in 1 mL of Ringer solution for haemolymph and 2 mL for ovaries and nerve chain. The resulting suspension was filtered through a 1.2 μm pore membrane to remove cell debris and host cell nuclei. A prior experiment conducted on 15 females from the source line (WXw) revealed that the number of bacteria per ng of total DNA (host + symbiont) differed among the three target tissues (see Figure S1). Therefore, the filtered solutions were diluted in Ringer solution to adjust the final number of bacteria at 2500 ± 300 per microliter for the trans‐infection batches used to describe *Wolbachia* infection dynamics, and at 1282 ± 205 bacteria per microliter for the two batches made to investigate bacterial persistence 200 days after injection. The variation in *Wolbachia* concentration between the two experiments stems from their differing timing, with the first conducted in January and the second in April. Since *Wolbachia* concentration (number/ng of DNA) can fluctuate based on host size and, consequently, age, this resulted in slight differences in *Wolbachia* concentration between the two trans‐infection experiments. Another prior experiment showed that the proportion of live bacteria in the different filtered tissue solutions was similar (LRT = 4.0279, *p* = 0.1335, proportion of live *Wolbachia* ± 95% CI, haemolymph = 0.815 ± 0.017, nervous chain = 0.875 ± 0.014, ovaries = 0.862 ± 0.037, see Figure S2 for a detailed protocol).

One μL of each filtrate was injected using a thin glass needle into the general cavity of each recipient individual through a small hole pierced at its posterior part (6th segment, slightly to the side). This injection protocol has not been shown to reduce the survival of recipient hosts (Le Clec'h et al. 2012; Pigeault et al. 2014). Each tissue filtrate from the three trans‐infection batches used to study *Wolbachia* infection dynamics (i.e., change in *Wolbachia* load over time, trans‐infection experiment 1) was injected into 21 recipient females of the WXa line, resulting in a total of 63 females transinfected per tissue filtrate. Then, on Days 20, 40 and 60 post‐injection, 6–7 recipient females per trans‐infection batch were randomly selected to quantify *Wolbachia* in the focal tissues (see Section 2.4). To study bacterial persistence in recipient host tissues (trans‐infection experiment 2), 10 females were injected with each tissue filtrate for each of the two trans‐infection batches. Two hundred days post‐injection, all surviving females were dissected to quantify *Wolbachia* in the three focal tissues (see Section 2.4). For each trans‐infection batch, control groups were created using the same protocol, but with female sources originating from the WXa line.

### 2.3 Survival Monitoring

The effect of *Wolbachia* injection on pill‐bug survival rate was monitored every 10 days as part of the study of the influence of *Wolbachia* tissue origin on the dynamics of early infection within the recipient host (trans‐infection experiment 1) and every 15–25 days as part of the study of *Wolbachia* tissue origin on the persistence of the bacterium in tissues (trans‐infection experiment 2). When individuals were harvested during the experiment to quantify *Wolbachia*, they were censored for the survival analysis (see Section 2.9).

### 2.4 Quantification of *Wolbachia* Load in Source and Recipient Host's Tissues

To compare *Wolbachia* load in different tissues of source line and recipient hosts, total DNA was extracted from the haemolymph, nerve chain and ovaries of individuals using standard protocols (Qiagen DNeasy 96 Blood and Tissue kit). For each sample, the purity of the extracted DNA (OD 260/280 and 260/230 nm ratios) was measured using a Nanodrop 1000 spectrophotometer (Thermofisher). To quantify *Wolbachia* load, we developed a fluorescent probe‐based quantitative PCR (qPCR) approach amplifying a *Wolbachia* locus and a reference host nuclear locus in the same reaction. We used primers wsp208f (5′‐TGG‐TGC‐AGC‐ATT‐TAC‐TCC‐AG‐3′) and wsp413r (5′‐TCG‐CTT‐GAT‐AAG‐CAA‐AAC‐CA‐3′) targeting the *Wolbachia* protein surface gene (*wsp*, Le Clec'h et al. 2012) and we designed primers amplifying a portion of the single‐copy nuclear gene encoding the mitochondrial leucine‐tRNA ligase of *A. vulgare* : TLeuF (5′‐TGT‐ACA‐CAT‐CGA‐GCA‐GCA‐AG‐3′) and TLeuR (5′‐AAA‐GAG‐GAG‐CGG‐AGA‐GTT‐TCA‐G‐3′) (Durand et al. 2023). The *Wolbachia wsp* double‐dye probe (5′‐TTG‐CAG‐ACA‐GTG‐TGA‐CAG‐CGT‐T‐3′) was labelled with hexachlorofluorescein (HEX) as a reporter at the 5′ end and Black Hole Quencher 1 (BHQ‐1) at the 3′ end. *A. vulgare* TLeu probe (5′‐ACG‐AAG‐TTC‐GCC‐CTG‐TTC‐TGG‐A‐3′) was labelled with 6‐carboxy‐fluorescein (FAM) as a reporter and BHQ‐1 as a quencher. The qPCR reactions were performed using Roche LIGHTCYCLER 480 with the following programme: 2 min at 50°C, 10 min at 95°C and 45 cycles, 15 s at 95°C and 1 min at 60°C. For each sample, two independent technical replicates were carried out and the mean cycle‐threshold value was calculated. *Wolbachia* load was then calculated relative to the reference gene (TLeu) using the delate CP method: 2^‐(CP(*wsp*) – CP(TLeu))^.

### 2.5 Genome Assembly of *Wolbachia* *w*VulC

To study the within‐host genetic diversity of *Wolbachia*, we first assembled the genome of the *w*VulC *Wolbachia* strain from the WXw line. Three female individuals were used for Oxford Nanopore Technologies (ONT) long‐read sequencing and one for Illumina short‐read sequencing. Total DNA from haemolymph, nerve chain and ovaries of each female was extracted using standard protocols (DNA used for ONT sequencing: Macherey‐Nagel NucleoBond HMW DNA kit, DNA used for Illumina sequencing: Qiagen DNeasy 96 Blood & Tissue Kit). The long‐read sequencing library was prepared using the SQK‐LSK109 Ligation Sequencing Kit (ONT) and sequenced with an R9.4.1 flow cell on a MinION Mk1B (ONT). Reads were basecalled with Guppy 6.3.8 (RRID:SCR_023196) using the SUP model. The short‐read library was prepared using the TruSeq Nano DNA Library Prep kit (Illumina) and sequenced on a HiSeq X (Illumina, paired‐end 2 × 150 bp).

ONT adapters were first trimmed with porechop 0.2.4 (https://github.com/rrwick/Porechop) and reads were filtered using NanoFilt 2.7.1 (minimum average quality: 7, minimum length: 1000 bp) implemented in NanoPack (De Coster et al. 2018). Reads were then assembled with Flye 2.8.3 (Kolmogorov et al. 2019) using default parameters. The entire assembly was first polished with the ONT reads using medaka 1.7 (https://github.com/nanoporetech/medaka, r941_min_fast_g507 model, 2 rounds). For assembly polishing using short reads, Illumina reads were mapped onto the assembly with bwa‐mem2 2.2.1 (Vasimuddin et al. 2019) and used for further polishing with Polypolish 0.4.3 (Wick and Holt 2022). This step was carried out twice. The largest assembled contig was reported detected as circular by the Flye assembler and had the expected length (~1.6 Mb) of the *w*VulC *Wolbachia* genome. We aligned this contig against a previous version of the genome (GenBank accession: ALWU00000000.1) with nucmer in MUMmer 4.0.0rc1 (Marçais et al. 2018), confirming that the contig corresponded to the *w*VulC strain. After mapping the short reads for a third time onto this contig, short variants were called with Freebayes 1.3.1 (Garrison and Marth 2012). Variants were then visually reviewed using the IGV genome browser (version 2.14.0) (Robinson et al. 2017). Eight of them, corresponding to errors, were kept. We corrected the genome sequence with these eight variants using the consensus command in BCFtools 1.17 (Danecek et al. 2021). Following the recommendations of Ioannidis (Ioannidis et al. 2007), the genome sequence was rotated to start at the *hemE* gene using the fixstart command of Circlator 1.5.5 (Hunt et al. 2015). The genome was annotated with the NCBI Prokaryotic Genome Annotation Pipeline (PGAP) 2022‐12‐13.build6494 (Haft et al. 2024; Li et al. 2021; Tatusova et al. 2016). A search of insertion sequence (IS) elements was carried out with digIS v1.2 (Puterová and Martínek 2021). Additionally, bacteriophage‐derived regions were detected using PHASTEST (Wishart et al. 2023). Results of these annotations were plotted with Circos v0.69‐9 (Krzywinski et al. 2009). The completeness of the genome and the gene annotation was assessed using BUSCO 5.3.2 (Manni et al. 2021) using the rickettsiales_odb10 dataset.

### 2.6 Whole‐Genome Resequencing and Small Variant Calling

Three *A. vulgare* females from the WXw line were used to study the genetic diversity of the different *Wolbachia* sub‐populations associated with the nerve chain, ovaries and haemolymph. Two of these females were sisters (names: F1_2015 and F2_2015). The matriline of the third one (F_1999) diverged from the matriline of the sisters for 18 generations (i.e., 18 years). Females were dissected and their tissues isolated individually. Total DNA of each biological sample was extracted using the Qiagen DNeasy 96 Blood & Tissue Kit. DNA purity and quantity were measured as described above. The haemolymph of two females could not be sequenced because it did not yield enough DNA. DNA libraries were prepared using the TruSeq Nano DNA Library Prep kit and sequenced on the Illumina HiSeq 2000 platform to generate 2 × 150 bp reads, by Genoscreen (Lille, France). Read quality was assessed with FastQC 0.11.9 (http://www.bioinformatics.babraham.ac.uk/projects/fastqc). Fastp 0.23.0 (Chen et al. 2018) was used to remove adapter contamination and systematic base calling errors. To mitigate misalignments of reads originating from the host genome, notably from *Wolbachia*‐derived nuclear insertions (Chebbi et al. 2019; Leclercq et al. 2016), reads were aligned using bwa‐mem2 (Vasimuddin et al. 2019) against a reference including our *w*VulC genome assembly, the assembly of the partial mitochondrial genome of *A. vulgare* (GenBank accession number: MF187614.1) (Peccoud et al. 2017) and the genome assembly of *A. vulgare* (accession number: GCA_004104545.1, Chebbi et al. 2019). PCR duplicates were marked with samtools markdup (v1.14). Read coverage along the *w*VulC genome was computed for each sample for adjacent 5 kb windows using mosdepth 0.2.9 (Pedersen and Quinlan 2018). For a given sample, normalised coverage for each window was calculated by dividing the coverage value by the median of all values in R (v. 4.2.1) (R Core Team 2022). SNPs and short indels were detected using Freebayes 1.3.1 (Garrison and Marth 2012), with a filter on the minimum fraction of observations supporting a variant (*F* = 0.03), disabling population priors (−k argument) and using an aggregate probability of observation balance between alleles (−a argument). Variants were then filtered using a custom R script. This script selected only variable positions for which the read total depth was over 200 and for which the alternative allele was supported by at least 10 reads within a tissue (*Wolbachia* genome coverage—mean ± SE: ovaries = 723.9 reads ±134.8, nerve chain = 769.8 ± 81.0, haemolymph = 533.7).

### 2.7 Small Variant Validation

As the *A. vulgare* lineage used in our experiments may contain nuclear inserts absent from the reference genome used for read mapping (Chebbi et al. 2019) the risk that sequences from *Wolbachia*‐derived nuclear inserts generated spurious SNPs may not have been eliminated. To exclude remaining spurious SNPs, we implemented a three‐step approach. (1) We checked that the sequences containing candidate SNPs were absent from uninfected individuals sequenced in previous studies, using all short‐read data generated by Chebbi et al. (2019) and Cordaux et al. (2021). These data constitute whole genome sequences of six pools of 10 males or females and five individuals, from three uninfected lineages. We aligned these short reads with bwa‐mem2 on the *w*VulC reference sequence. Then, we defined a 300‐bp window around the position of each variant that passed the first filter (starting 150 bp before the site) and excluded variants in windows containing at least two reads from uninfected individuals. (2) To further check that each remaining variable region was absent from uninfected individuals, we then designed PCR primers to amplify 120–250 bp targets encompassing the focal variant. These primers were tested on two females from the recipient lineage (i.e., uninfected by *Wolbachia*), on two females from the source line (i.e., infected by *Wolbachia*) and on two of their brothers (not infected by *Wolbachia*, since infection induces feminization). If the primers amplified DNA from at least one non‐infected host, then the variant was eliminated. (3) Finally, we checked whether sequencing reads carrying non‐reference SNP alleles represented sequences that were exceedingly divergent from the reference strain (e.g., insertions of *Wolbachia* DNA into the host genome or a related bacterium). To do so, we automatically analysed every candidate SNP via a script that parses the alignment (.bam) files of reads generated in this experiment. This script records the proportion of clipped reads, the proportion of read pairs that were aligned in proper pairs and the number of mismatches between reads and the reference (excluding the SNP itself). The script then performs Fisher exact tests to evaluate if these proportions significantly differ between reads carrying the reference allele and reads carrying the alternative allele. Any SNP for which the *p*‐value of any test was below 5% (after false discovery rate correction) was excluded. Finally, the sequencing depth around SNPs that had passed all filters was averaged over 100‐bp windows, via samtools depth and plotted to check for anomalies, mainly the presence of collapsed repeated regions not fully resolved in the *Wolbachia* reference genome assembly.

To predict the effect of retained SNPs and indels on proteins coded by annotated genes, we used SNPeff 5.2c (Cingolani et al. 2012). Functional domains in these proteins were annotated using the InterProScan online service (http://www.ebi.ac.uk/interpro/, database release 100.0, Paysan‐Lafosse et al. 2023). Finally, to determine whether the *Wolbachia* variant(s) validated by our SNP filtration procedure arose during the lifetime of the focal individuals or were inherited maternally, we searched for these variants in their maternal lineage using an amplification‐refractory mutation system (see Section 4 and Figure S4 for details of the protocol). Since the animals did not reproduce, we were unable to study transmission of this variant to offspring.

### 2.8 Detection of Macromutation

After identifying SNPs within the different *Wolbachia* subpopulations, we investigated whether these subpopulations might also differ through the presence of macromutations (deletions, duplications and insertions ≥ 50 bp, as well as inversions and translocations). To do this, we used programmes Delly (Rausch et al. 2012) and Lumpy (Layer et al. 2014), as both tools can detect multiple types of structural variants (SVs) from Illumina reads and can be easily run directly from BAM files. Only a single SV was detected and it was identified by both tools. This genomic variation was visualised using IGV (version 2.14.0). To confirm the nature of the detected macromutation, we realigned the reads from the sequencing of the different *Wolbachia* subpopulations using bwa‐mem2; this time with the unpaired reads option enabled.

### 2.9 Statistical Analyses

Statistical analyses were carried out using the R software (R Core Team 2022). The influence of the tissular origin of the injected *Wolbachia* on *A. vulgare* survival rate was evaluated using the ‘survreg’ function with either exponential or Weibull errors distribution (‘survival’ package, Therneau et al. 2024). The model included survival as a response variable and infection status (i.e., injected with *Wolbachia*‐infected or uninfected tissue filtrate), tissue origin of the injected filtrate and experimental block as explanatory variables. The influence of *Wolbachia* tissue origin on its ability to colonise and infect different focal tissues of recipient hosts over time was assessed using a generalised linear mixed model (GLMM). Log‐transformed relative quantification (RQ) values representing the per‐host cell *Wolbachia* dose were fitted as a response variable, assuming normal error distribution. The tissular origin of *Wolbachia*, the focal tissue of recipient injected hosts and the day post injection were used as explanatory variables. The quadratic term day post‐injection was added to assess whether it significantly improved the model fit. Individual IDs nested in experimental block were specified as random‐effect variables in the model. To compare *Wolbachia* loads measured in the tissues of recipient hosts 6 months after experimental infection with that measured in the same tissues from females of the same age but naturally infected by *Wolbachia* (from the WXw line), we ran, for each tissue, a linear model with log‐transformed RQ values fitted as a response variable and the origin of the individuals (i.e., naturally infected or experimentally infected) as an explanatory variable.

Maximal models including all higher‐order interactions were simplified by sequentially eliminating non‐significant terms and interactions to establish a minimal model (Crawley 2012). The significance of the effects of explanatory variables was established using a likelihood ratio test (LRT) (Crawley 2012), whose statistics approximately follows a Chi‐square distribution (Bolker 2008) or an *F* test. The significant LRT or *F* value given in Section 3 is for the minimal model, whereas non‐significant values correspond to those obtained before the deletion of the variables from the model. A posteriori contrasts were carried out by aggregating factor levels together and by testing the fit of the simplified model using a likelihood‐ratio test (Bolker 2008; Crawley 2012).

## 3 Results

### 3.1 *Wolbachia* Infection Does Not Significantly Influence Host Survival Rate

**FIGURE 1:**
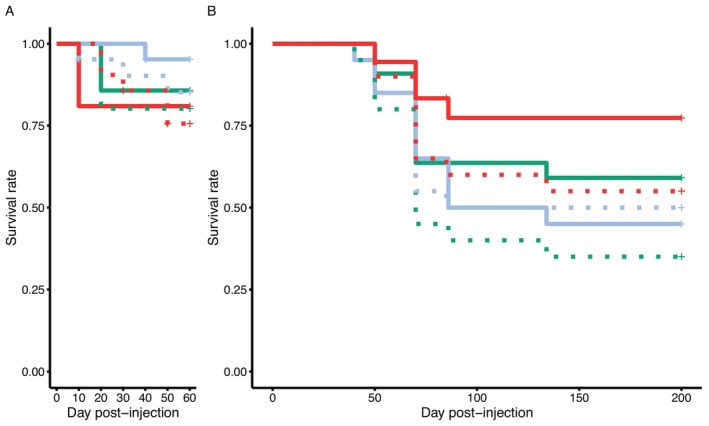
Survival rate of *Armadillidium vulgare* following *Wolbachia* (*w*VulC) trans‐infection. Effect of tissue origin of injected *Wolbachia* on survival rate of recipient hosts monitored (A) every 10 days from Days 0 to 60 post‐injection and (B) every 15–20 days from Days 0 to 200 post‐injection. Solid lines: recipient hosts injected with crushed filtered tissue from animals naturally infected by *Wolbachia w*Vulc. Dotted lines: control individuals injected with crushed filtered tissue *from A. vulgare* not infected by *Wolbachia*. The three colours correspond to the tissue of origin of *Wolbachia*. Green: nerve chain, orange: ovaries, blue: haemolymph.

Under the assumption of both exponential errors and non‐constant hazard, no effect of *Wolbachia* tissue origin on the survival rate of *A. vulgare* during the early stage of infection (from Days 0 to 60, trans‐infection experiment 1) was detected (exponential errors: LRT = 0.600, *p* = 0.740, Weibull errors: LRT = 0.602, *p* = 0.741, Figure 1A). Overall, the injection of *Wolbachia*, irrespective of its tissue origin, was not shown to influence the survival of individuals (exponential errors: LRT = 1.040, *p* = 0.308, Weibull errors: LRT = 1.039, *p* = 0.307). Sixty days after injection, the survival rate of infected individuals was 0.87 (±95% CI, ±0.79–0.96), compared to 0.80 (±0.71–0.91) for control animals.

In the trans‐infection batches used to investigate the influence of *Wolbachia* tissue origin on its ability to persist in recipient host tissue (trans‐infection experiment 2), the survival rate of animals was also not significantly influenced by *Wolbachia* tissue origin (exponential errors: LRT = 2.65, *p* = 0.265, Weibull errors: LRT = 2.88, *p* = 0.236) or even just by the fact of being infected by the bacteria (whatever the origin of the tissue, exponential errors: LRT = 2.22, *p* = 0.136, Weibull errors: LRT = 2.45, *p* = 0.117, Figure 1B). The survival rate of infected individuals 200 days after the experimental infection was 0.59 95% CI: 0.48–0.74 and 0.47 95% CI: 0.36–0.61 for infected and control animals, respectively. Considering that *A. vulgare* typically lives for about 3 years in laboratory conditions and that the individuals were approximately 2 years old at the start of the experiment, the nearly 50% decline in survival over 200 days is therefore not unexpected.

### 3.2 The Tissue Origin of *Wolbachia* Influences Its Early Infection Dynamics in Recipient Host Tissues (Trans‐Infection Experiment 1)

**FIGURE 2:**
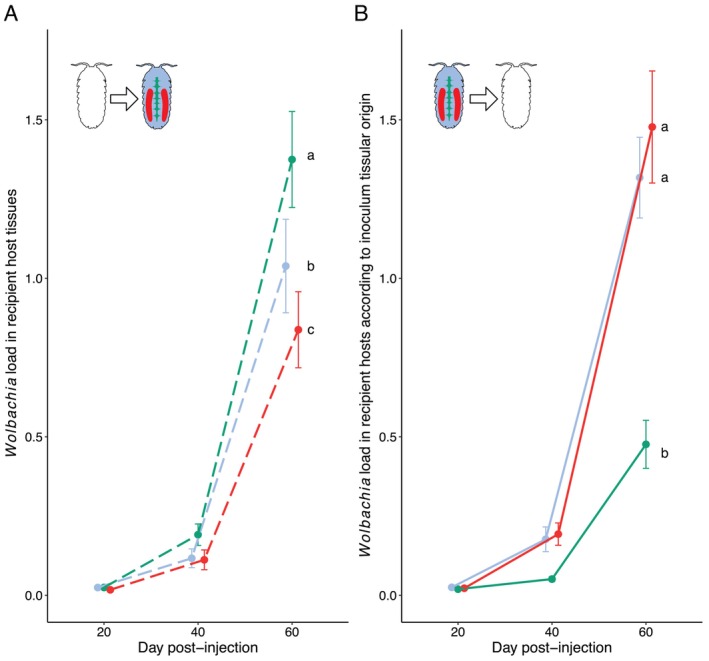
Infection dynamics of *Wolbachia* (*w*VulC) in *Armadillidium vulgare* from day 0 to day 60 post‐injection. (A) Colonisation of recipient host tissues by *Wolbachia* 20, 40 and 60 days after transinfection. Here, the tissue origin of the injected *Wolbachia* is not considered. Green, blue and red colours respectively represent *Wolbachia* loads in the nerve cord, hemolymph and ovaries. (B) Influence of the tissue from which bacteria were extracted in naturally infected hosts on infection dynamics (change in *Wolbachia* load over time) in recipient host tissues (nerve cord, hemolymph and ovaries combined) at 20‐, 40‐ and 60‐days post‐injection. Colour codes follow (A), but for tissues of the source host. Infection dynamics not connected by the same letter are significantly different (*p* < 0.05). Note that there was no significant interaction between the colonised recipient tissue and the original *Wolbachia* tissue, but the figure representing this interaction is presented in Figure S3.

After injection, the presence of *Wolbachia* was detected by qPCR in the three tested tissues of all recipient hosts, confirming its ability to colonise a new host. *Wolbachia* load increased over time (post‐injection day: LRT = 16.239, *p* < 0.0001) and fitting the quadratic term (day_post_injection^2^) strongly improved model fit (LRT = 32.181, *p* < 0.0001), suggesting that *Wolbachia* burden was more accurately modelled by an accelerated polynomial function of day post‐injection (Figure 2A). The load of *Wolbachia* in tissues started very low on Day 20 post‐injection, then rose slightly between Days 20 and 40, to drastically increase between Days 40 and 60 post‐injection. Although the pattern of infection in the different recipient tissues was overall similar, *Wolbachia* load was significantly influenced by the interaction between the colonised recipient tissue and the day post‐injection (LRT = 74.309, *p* < 0.0001). The dynamics of infection within the nerve chain (i.e., change in *Wolbachia* load over time) were significantly different from those observed in the haemolymph and ovaries, leading to a higher bacterial load in nerve tissues 60 days post‐infection (LRT = 57.228, *p* < 0.0001, LRT = 122.240, *p* < 0.0001, respectively, Figure 2A). The dynamics of infection were also significantly different between haemolymph and ovaries (LRT = 17.771, *p* = 0.0013, Figure 2A), as bacterial load was lower in ovaries at the end of monitoring (mean RQ ± SE, nerve chain = 6.582 ± 1.596, haemolymph = 4.864 ± 1.612, ovaries = 2.533 ± 0.594, Figure 2A). *Wolbachia* infection dynamics were also influenced by the tissue origin of the injected bacteria (interaction between days post‐injection and tissue origin: LRT = 24.205, *p* < 0.0001). Whether the bacteria came from the haemolymph or ovaries of naturally infected animals did not significantly affect the colonisation dynamics of *Wolbachia* in the injected recipients (LRT = 0.647, *p* = 0.723, Figure 2B). However, bacteria from the nerve chain colonised recipient hosts less rapidly than bacteria from other tissues (nerve chain versus haemolymph: LRT = 28.981, *p* < 0.0001, nerve chain versus ovaries: LRT = 33.64, *p* < 0.0001). At 60 days post‐injection, the *Wolbachia* load was on average more than twice as high when the bacteria came from the ovaries and haemolymph than when they came from the nerve chain of infected source hosts (mean RQ ± SE, ovaries = 8.191 ± 2.18, haemolymph = 5.26 ± 1.077, nerve chain = 0.899 ± 0.197, Figure 2B). However, there was no significant interaction between the colonised recipient tissue and the *Wolbachia's* original tissue, suggesting that bacteria originating from a tissue do not colonise that same tissue more effectively in recipient hosts (LRT = 6.957, *p* = 0.138, see Figure S3).

### 3.3 The Tissue Origin of *Wolbachia* Does Not Influence Significantly Its Persistence in Recipient Host Tissues (Trans‐Infection Experiment 2)

**FIGURE 3:**
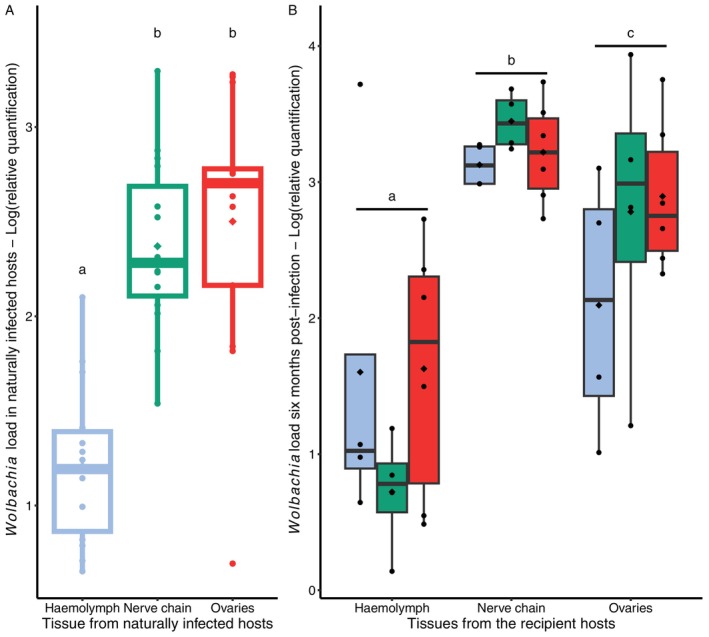
*Wolbachia* (*w*VulC) load in tissues of *Armadillidium vulgare* (A) naturally infected by *Wolbachia* or (B) in tissues of experimentally infected hosts 6 months post‐injection. (A) Bacterial load in tissues of naturally infected females (source line). *Wolbachia* load was measured in the haemolymph (blue), nerve chain (green) and ovaries (red) of 15 females from the source line. The colour of the boxplot borders corresponds to the tissue in which *Wolbachia* was quantified in naturally infected hosts (green: nerve chain, red: ovaries, blue: haemolymph). (B) Bacterial load in tissues of recipient hosts injected with *Wolbachia* 6 months ago. The *x*‐axis represents the tissues of the recipient hosts. The coloured boxplots indicate the tissue origin of *Wolbachia* (green: nerve cord, red: ovaries, blue: hemolymph). Boxes above and below the medians (horizontal lines) show the first and third quartiles, respectively. Black diamonds represent the means. Levels not connected by the same letter are significantly different (*p* < 0.05).

*Wolbachia* densities at 200 days after infection were significantly different between tissues from the recipient hosts (LRT = 39.224, *p* < 0.0001, Figure 3A). Bacterial load was the highest in the nerve chain and the lowest in the haemolymph (contrast analyses, haemolymph versus nerve chain: LRT = 38.133, *p* < 0.0001, haemolymph versus ovaries: LRT = 20.823, *p* < 0.0001, nerve chain versus ovaries: LRT = 6.969, *p* = 0.008, Figure 3A). The bacterial load in the ovaries and haemolymph of recipient females was similar to those observed in the same tissues of females naturally infected by *Wolbachia* (source lineage, *F* = 0.184, *p* = 0.672, *F* = 0.268, *p* = 0.609, respectively, Figure 3A,B). In contrast, bacterial load in the nerve chain of experimentally infected females was significantly higher than that observed in naturally infected individuals (*F* = 38.089, *p* < 0.0001, Figure 3A,B).

Contrary to what was observed during the early phase of infection, there was no effect of the tissue origin of *Wolbachia* on the bacterial load in recipient tissues at 200 days post‐injection (LRT = 0.368, *p* = 0.832). Similarly, *Wolbachia* load in recipient tissues was not influenced by the interaction between the colonised tissue and the tissue origin of the injected bacteria (LRT = 9.539, *p* = 0.059).

### 3.4 Complete Genome Assembly of the *w*VulC *Wolbachia* Strain

**FIGURE 4:**
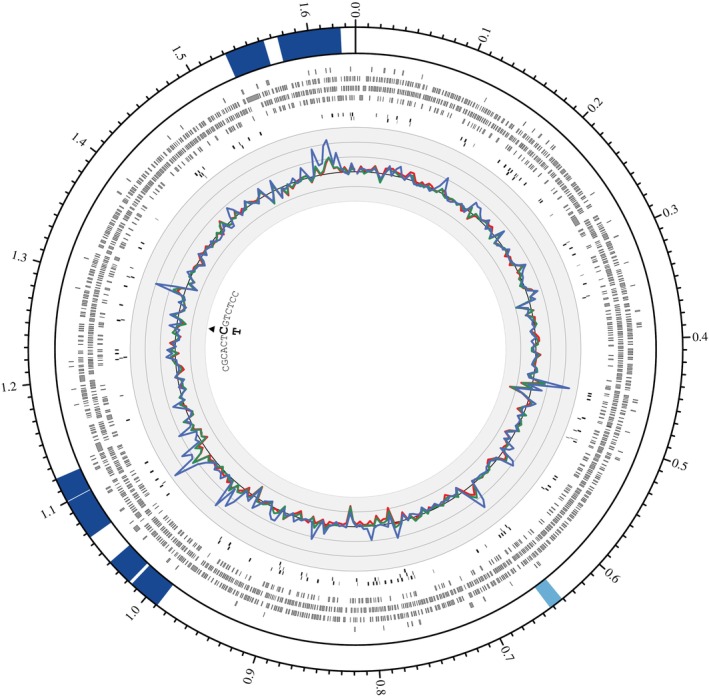
Circos plot of the wVulC *Wolbachia* genome. Prophage‐derived regions are indicated inside the genome ideogram (outer track), with their colour corresponding to the score attributed by PHASTEST (dark blue: intact, light blue: questionable). Ticks on the ideogram are separated by 10 kb intervals and indicated values are in Mb. Predicted protein‐coding genes are represented by grey rectangles (second track), and Insertion Sequence elements by black rectangles (third track). In the fourth track, the normalised read coverage for 5 kb adjacent windows (averaged over recipient individuals) is represented for the different resequenced tissues: ovaries (red), nerve chain (green) and haemolymph (blue). The black line corresponds to a mean normalised coverage of one and grey lines are separated by 0.1 unit. Black triangle indicates the position of the detected variant and the bold letter (C and T) correspond to the two alleles.

Using ONT reads, a complete circular genome sequence (1,638,144 bp) of the *w*VulC *Wolbachia* strain was assembled. The estimated completeness of the assembly was very high, with 99.8% of the expected BUSCO genes found in the sequence (*C*: 99.8% [*S*: 99.5%, *D*: 0.3%], *F*: 0.0%). The annotation of the genome resulted in 1390 protein‐coding sequences and 208 pseudogenes (Figure 4). The *w*VulC genome contained 138 IS elements that are larger than 150 bp, representing 8.45% of the total length of the genome. This high proportion of repeated sequences for a bacterium is similar to that of other *Wolbachia* strains (Cerveau et al. 2011). We also identified seven prophage regions (six intact and one questionable region, following PHASTEST scoring criteria), representing 12.1% of the *w*VulC genome.

### 3.5 Whole‐Genome Resequencing and Variant Calling Reveal a Relative *Wolbachia* Genome Stability

The small variant calling performed using the whole‐genome resequencing of tissues from three females naturally infected by *Wolbachia* (i.e., source lineage) detected 641 SNPs and short indels. However, for 610 of these apparent variants, we also detected reads in the 300 bp windows placed around each SNP in the resequenced genome of several *Wolbachia*‐uninfected *A. vulgare* lineages (validation procedure, step 1), meaning that these variants probably correspond to *Wolbachia* sequences inserted into the host nuclear genome. Of the remaining 31 SNPs and small indels, 7 were excluded because their regions were PCR‐amplified in males not infected with *Wolbachia* (infection status confirmed by qPCR, validation procedure step 2). Finally, in the last step of variant validation, we excluded 23 variants due to anomalies detected, such as a high proportion of clipped reads and/or a high number of mismatches between reads and reference. Overall, only 0.15% (one SNP) of the originally detected variants were finally validated. This unique SNP was observed in a single individual (F_1999). The frequency of this variant varied slightly among the three tissues (Table 1). The mutation affected a protein‐coding gene, with a non‐synonymous substitution (Table 1, Figure 4). The putative protein did not have any recognised functional domain. The variant was not detected in the F_1999 individual's sisters and was also absent from all females tested in the maternal line (traced back six generations, see Appendix 1 in the Supporting Information).

**TABLE 1:** Nucleotide difference between *Wolbachia* from three tissues of naturally infected *Armadillidium vulgare* .

Genome position	Individual	Tissue	Frequency	Type of change	Nucl. change	AA change	ORF effect	Gene name	Functional domains
1,269,492	F1_2015	NC	0	Substitution	C → T	Arg340Gln	Missense variant	ABOJ95_001275	No recognised functional domain
		OVA	0						
	F2_2015	NC	0						
		OVA	0						
	F_1999	HAEM	0.466						
		NC	0.584						
		OVA	0.239						

Abbreviations: AA: amino acid; Arg: arginine; Gln: glutamine; HAEM: haemolymph; NC: nerve chain; Nucl: nucleotide; OVA: ovaries.

**FIGURE 5:**
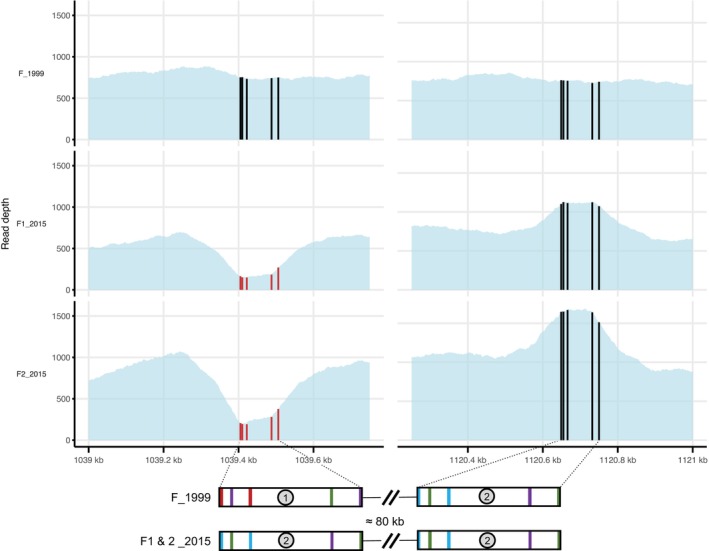
Regions involved in the suspected gene conversion event in the two sisters F1_2015 and F2_2015. The first part of the figure shows sequencing depth across region (1) and region (2), which are separated by approximately 80 kb. In females F1_2015 and F2_2015, a decrease in coverage is observed in region (1), followed by an increase in region (2). In contrast, sequencing depth remains stable across both regions in female F_1999. Black bars in the F_1999 track indicate five nucleotide differences between two highly conserved ~100 bp sequences in region (1) and region (2). In females F1_2015 and F2_2015, these positions are shown as red marks in region (1), indicating that the nucleotides are identical to those in region (2) and thus differ from both the reference genome and F_1999. The second part of the figure displays a close‐up of the two ~100 bp genomic regions. Different colours highlight the nucleotide differences distinguishing sequences in region (1) and region (2) in F_1999 and in the reference genome (red = T, purple = G, green = A, blue = C).

The SVanalyses did not reveal any SV specifically associated with a *Wolbachia* subpopulation from a particular host tissue. However, both tools—Delly and Lumpy—identified a macromutation at position 1,039,540 in the *Wolbachia* subpopulations from the nervous chain and ovaries of females F1_2015 and F2_2015. The genomic variation detected, which is absent from all tissues of female F_1999, was classified by both tools as a duplication. However, visual inspection of this region in IGV clearly shows that it is not a macromutation but rather a gene conversion event that likely occurred in the maternal lineage of the two sisters, F1 and F2_2015. Indeed, we observe a very sharp drop in coverage in both sisters over a 400 bp region, followed by an increase in coverage over a region of identical size some 80 kb further on (Figure 5).

The *Wolbachia w*VulC genome contains two genes located approximately 80 kb apart, which share high sequence homology (92.16%; see Figure 5). These genes encode proteins belonging to the Helix‐turn‐helix family (GenBank: XCA36066.1). In female F_1999, as well as in the reference genome used for read alignment, a sequence of 104 nucleotides—covering the 3′ end of the gene and extending 50 bases downstream—shows strong homology (97%) between the two loci (hereafter referred to as Region 1 and Region 2, Figure 5). Only five nucleotide differences distinguish these two regions. In contrast, in F1_2015 and F2_2015, the two 104‐nucleotide segments are identical (100% sequence identity) and both match Region 2 of the reference genome. Since the Region 1 sequences in F1_2015 and F2_2015 differ from those in the reference (and F_1999), most of the reads aligned to Region 2, causing a notable increase in coverage at this locus (Figure 5). Nevertheless, a subset of reads from F1_2015 and F2_2015 still aligned to Region 1. This is due to constraints in paired‐end alignment (linked to insert size). Notably, all the reads mapped to Region 1 in these individuals carry one or more nucleotides that are diagnostic of Region 2 in the reference. When we realigned the reads with bwa but without the pair‐end option, we observe that, for females F1_2015 and F2_2015, no more reads have been aligned to Region 1; all aligned to Region 2, leading to a strong increase in coverage (see Figure S5).

## 4 Discussion

Experimental trans‐infections showed that *Wolbachia*'s ability to colonise a new host depended on their tissue of origin. Bacteria originating from the nerve chain of naturally infected hosts colonised the tissues of recipient hosts significantly more slowly than bacteria originating from the ovaries or the haemolymph. Genome resequencing did not reveal genetic variation (SNPs or structural mutations) related to this phenotypic variation. At the same time, regular monitoring of injected individuals over a period of 6 months shed light on the influence of *Wolbachia* on the survival rate of recipient hosts and did not reveal any difference in virulence between *Wolbachia* sub‐populations. In addition, our results corroborate a previous report showing a similar survival rate between trans‐infected and un‐infected animals (Le Clec'h et al. 2012). As the source and recipient lines belonged to the same field‐collected *A. vulgare* population, they should be ecologically similar for *Wolbachia*. Low virulence is therefore expected, as for most vertically transmitted endosymbionts transferred between closely related hosts (Le Clec'h, Chevalier, et al. 2013, Le Clec'h, Raimond, et al. 2013). Nevertheless, a slight difference was observed between the two experimental blocks (transinfection experiment 1 and transinfection experiment 2), with higher mortality at the beginning of transinfection experiment 1 compared to experiment 2. We have no clear explanation for this difference other than the fact that the two experiments were initiated at different times of the year (February and April, respectively).

Colonisation of recipient female tissues was relatively fast, with a threefold increase in *Wolbachia* load between the second and sixth month after infection. At the end of the experiment, the bacterial load in the tissues of the recipient females was similar to, or even slightly higher than, that observed in the tissues of naturally vertically infected females. Nevertheless, although the infection progressed rapidly, the dynamics of *Wolbachia* proliferation depended on the tissue from which the bacteria originated. Bacterial growth rates are well known to vary substantially with the type and availability of nutrients in their environment (Keiblinger et al. 2010; Liu et al. 2005). The lower growth rate of bacteria from the nerve chain could therefore be explained by the fact that in naturally infected hosts, this tissue is less favourable to the development of *Wolbachia* (e.g., poorer in resources). If so, bacteria from the nerve chain could be subject to growth‐limiting stress at the time of their horizontal transfer, which would explain their lower replication rate within the recipient host at the start of infection. However, the nerve chain was the recipient host tissue the most rapidly colonised by *Wolbachia* after trans‐infection and the one showing the highest bacterial load at each sampling point irrespective of the tissue origin of the bacteria. As well as showing no tropism towards the tissue of origin, this result suggests that nervous tissue is actually favourable to the development of *Wolbachia*.

Alternatively, the lower growth rate from bacteria originating from the nerve chain could be adaptive. Classic theory for the evolution of virulence is based on a trade‐off between parasite growth, transmission and host survival, which predicts that higher growth increases not only transmission but also virulence (Alizon et al. 2009; Frank 1996). *Wolbachia* transmission is essentially maternal (Turelli et al. 2018; Werren et al. 2008), but cases of non‐maternal transmission have been observed *in natura* (Durand et al. 2024; Sanaei et al. 2021) and in the laboratory (Le Clec'h, Chevalier, et al. 2013; Le Clec'h, Raimond, et al. 2013; Rigaud and Juchault 1995). In a tissue not directly involved in parasite transmission, we predicted that virulence should constrain the parasite growth to low levels. Our results corroborate this prediction, as *Wolbachia* from the nerve chain of *A. vulgare* should not be transmissible—except maybe in rare cases of cannibalism and predation (Le Clec'h, Chevalier, et al. 2013; Le Clec'h, Raimond, et al. 2013)—and may prove detrimental to the host if they proliferate. Uncontrolled colonisation of nerve cells has indeed been shown to alter host behaviour in several *Wolbachia*‐host pairs (Bi and Wang 2020; Le Clec'h et al. 2012) and to lead to severe tissue degeneration and premature host death (Kosmidis et al. 2014; Le Clec'h et al. 2012; Min and Benzer 1997; Strunov and Kiseleva 2016). In tissues that allow parasite transmission, the balance between *Wolbachia* growth and host fitness should favour higher growth rates. In terrestrial isopods, both ovaries and haemolymph play a key role in both intra‐ and inter‐host dissemination of *Wolbachia*. Ovaries are the route for vertical transmission and haemolymph, in addition to participating in the dissemination of the bacteria across host tissues (Braquart‐Varnier et al. 2015; Sanaei et al. 2021), may also be involved in horizontal transfer via contact between injured individuals (Rigaud and Juchault 1995).

The convergence of *Wolbachia* growth rates observed six months post‐injection suggests that the early phenotypic variability detected two months post‐infection stems from phenotypic plasticity. This interpretation is further supported by the absence of recurrent genomic variations associated with tissue types in naturally infected individuals from the source lineage. Indeed, a single SNP was detected in one female (F_1999) and was found in all three tissue types, with allele frequencies ranging from 24% to 58% depending on the tissue. This mutation, located in a gene coding for a protein of unknown function, leads to an amino acid substitution. Although we currently have no information on the phenotypic impact of this mutation, amino acid substitution may have strong phenotypic effects in bacteria (e.g., Abdelaal et al. 2009; Bacigalupe et al. 2019). The variant, although predominant in the nerve chain, was present at lower frequencies in the ovaries. Nevertheless, over a quarter of the *Wolbachia* population in this tissue carried the mutation. This suggests that some of the female's oocytes may have been colonised by the variant and that, despite the suspicion of a strong bottleneck, it could have passed it on to its offspring (Chrostek and Teixeira 2015). However, this hypothesis could not be verified in our study, as the female was sacrificed before reproducing. What is clear is that the absence of this SNP within the individual's maternal lineage strongly suggests that this *Wolbachia* variant arose during its lifetime. The emergence of *Wolbachia* variants within just a few host generations or even during a host's lifetime has been documented (Chrostek and Teixeira 2018; Martinez and Sinkins 2023; Namias et al. 2024; Newton and Sheehan 2015). However, most reported variants were associated with structural genomic changes rather than point genomic mutations (e.g., Chrostek and Teixeira 2018; Namias et al. 2024), which is why we also looked for this type of macromutation in our dataset.

The SV analyses did not reveal any macromutation associated with a *Wolbachia* subpopulation associated with a specific tissue. On the other hand, we characterised what appears to be a gene conversion event in the two sister females (F1_2015 and F2_2015). Gene conversion is a well‐documented mechanism of homologous recombination in bacteria that contributes to genetic diversity, DNA repair and the homogenisation of gene families (Santoyo and Romero 2005; Paulsson et al. 2017). In *Wolbachia*, horizontal gene transfer and recombination have been extensively reported (Werren and Bartos 2001; Hill et al. 2022; Baldo et al. 2006; Ellegaard et al. 2013), but gene conversion events remain less thoroughly explored (see Cordaux 2009; Jia et al. 2024). Yet, genomic analyses suggest that gene conversion may play a role in the evolution of the *Wolbachia* genome (Cordaux 2009; Leclercq et al. 2011). Here, it is the 3′ region of a gene involved in gene expression regulation that is affected. Our experimental design does not allow us to determine whether this gene conversion has any effect on the phenotype of the *Wolbachia* strain present in the F1 and F2_2015 females. The only thing we observe is that the bacterial load in the three individuals studied does not appear to differ (see Figure S6).

Although we identified inter‐individual genomic polymorphism—specifically, a SNP and one gene conversion event—our findings align with previous reports from other *Wolbachia*/host systems and support the overall genomic stability of *Wolbachia* (Dainty et al. 2021; Huang et al. 2020; Ross et al. 2022; Trouche et al. 2024). This stability is likely due to a low mutation rate as well as a strong bottleneck at the time of transmission. However, because *Wolbachia* is an obligate intracellular endosymbiont, direct estimates of its mutation rate and effective population size remain extremely limited. To our knowledge, only one study has estimated the substitution rate of *Wolbachia* in *Drosophila*, reporting a relatively low rate compared to other bacteria (Richardson et al. 2012; Biek et al. 2015). Other infectious agents responsible for systemic infections and exhibiting higher mutation rates could serve as valuable biological models to study the ability of parasites to adapt to the tissue microenvironments they colonise.

To conclude, our study reveals that within‐host environmental heterogeneity can lead to diverse phenotypes in the most widespread bacterial endosymbiont in animals, *Wolbachia*. This variability did not appear to result from genomic variation, but from phenotypic plasticity. To explore this further, it would be relevant to investigate the mechanisms underlying the observed expression plasticity. A logical first step would involve comparative transcriptomic analyses across distinct *Wolbachia* subpopulations. In a second phase, expression polymorphism could be examined using spatially targeted microproteomics. These two approaches could help identify the metabolic pathways underlying the expression of distinct phenotypes. The modulation of gene expression leading to distinct bacterial phenotypes within different host tissues may be mediated by the epigenetic state of the bacteria. Indeed, the formation of epigenetic lineages has been shown to enable bacterial populations to adapt to hostile or fluctuating environments and may modulate their interactions with eukaryotic host cells (Casadesús and Low 2006; Sánchez‐Romero and Casadesús 2020; Ni et al. 2012). Finally, from a methodological point of view, we showed that the detection of variants in endosymbiont populations requires considerable caution. Our conservative approach to detect SNP led us to exclude more than 99.85% of the initially called variants. We recommend the use of a rigorous step‐by‐step approach to eliminate spurious genetic variants caused by the presence of endosymbiont genomic sequences inserted into the host genome.

## Author Contributions

R.P. and R.C. conceived and designed the experiments. R.P., R.J., M.P. and M.R. performed the trans‐infection experiments. R.P., T.U. and W.A. performed the preliminary experiment. R.P. and C.D. prepared the samples for resequencing and C.D., T.B. and T.U. carried out the PCRs. D.O. performed *Wolbachia* genome sequencing. Y.D. assembled the *Wolbachia* genome. R.P., Y.D., B.M. and J.P. analysed the data. R.P. wrote the first draft of the manuscript and all authors contributed substantially to revision.

## Funding

This work was supported by Agence Nationale de la Recherche (ANR‐21‐CE02‐0004 and ANR‐20‐CE02‐004).

## Ethics Statement

The authors have nothing to report.

## Conflicts of Interest

The authors declare no conflicts of interest.

## Supporting information


**Data S1:** emi470286‐sup‐0001‐Supinfo.docx.

## Data Availability

The R scripts and data supporting the conclusions of this article are available on the Figshare data repository (https://figshare.com/s/b1f84643b65b4f897c54). To ensure the stability and reliability of the command‐lines used for genome assembly and SNP calling, we have included all necessary information and options in the GitHub repository (https://github.com/UMR‐CNRS‐7267/Wolbachia_endosymbiont_heterogeneity_paper). We have taken proactive measures to address potential volatility on GitHub by uploading a compressed archive of this GitHub methodology to the Figshare data repository. Raw sequencing data have been deposited in GenBank (wVulC genome assembly: BioProject PRJNA1116085; Wolbachia resequencing: PRJNA1117639).
