# Supplementary material for: Within‐Host Environmental Heterogeneity Is Associated With Phenotypic but Not Genomic Diversity in Wolbachia Endosymbionts
Source: Environ Microbiol Rep. 2026 Feb 16;18(1):e70286. doi: 10.1111/1758-2229.70286 (PMC12908729; doi:10.1111/1758-2229.70286)
Supplement: Supplementary file 1 — Data S1: emi470286‐sup‐0001‐Supinfo.docx. [file EMI4-18-e70286-s001.docx]

Tissue heterogeneity is associated with phenotypic but not genomic diversity in *Wolbachia* endosymbionts

Romain Pigeault^1*^, Yann Dussert^1^, Raphaël Jorge^3,4^, Theo Ulve^1^, Marie Panza^1^, Maryline Raimond^1^, Carine Delaunay^1^, Willy Aucher^1^, Thierry Berges^1^, David Ogereau^5^, Bouziane Moumen^1^, Jean Peccoud^1†^, Richard Cordaux^5†^

^1^ Université de Poitiers, CNRS, EBI, UMR 7267, Poitiers, France

^2^ Génomique Métabolique, Genoscope, Institut François Jacob, CEA, CNRS, Univ Evry, Université Paris-Saclay, Evry, 91057, France

^3^ Université Lyon 1, CNRS, VetAgroSup, Laboratoire de Biométrie et Biologie Evolutive, UMR 5558, Villeurbanne, France

^4^ INSA Lyon, INRAE, Biologie fonctionnelle, insectes et interactions, UMR203, Villeurbanne, France

^5^ Université Paris-Saclay, CNRS, IRD, UMR Évolution Génomes Comportement Écologie, 91190 Gif-sur-Yvette, France

**Supplementary materials**

# *Wolbachia* quantification in tissues of naturally infected (*A. vulgare*)

1. Estimation of the proportion of live *Wolbachia* in filtered tissue solutions injected into recipient hosts.
2. Figure S3: No interaction between the colonized recipient tissue and the *Wolbachia*'s original tissue
3. Screening for variants in maternal lines
4. IGV screenshot showing the alignment of reads realigned with bwa but without the pair-end option
5. Relationship between *Wolbachia* relative quantification in host’s tissues estimated by qPCR and using Illumina reads (Figure S5).

# *Wolbachia* quantification in tissues of naturally infected *A. vulgare*

Previous studies have shown that the density of *Wolbachia* in the tissues of naturally infected hosts was different between tissues (e.g., Le Clec'h et al 2017). Here, we aimed to inject similar quantities of bacteria in recipient hosts, independently of their tissue origin. To achieve this, it was necessary to adjust the concentration of bacteria per ng of DNA in the different tissue solutions before injection into recipient hosts. To calculate the dilution factors of the different solutions, we first estimated the *Wolbachia* density per ng of DNA in the nerve chain, ovaries and haemolymph of 15 females from the source line (WXw). Absolute quantification of *Wolbachia* was estimated by quantitative PCR as described in the “Materials and methods” section of the main text. The gene copy number of *wsp* was then estimated by calculation in reference to a standard curve. The total DNA quantity (i.e. host+*Wolbachia*) of each sample, measured by fluorescence-based Qubit quantitation assays (Invitrogen™ Qubit™ Fluorometer), was used to normalize *wsp* gene copy number. The results are thus given in number of *wsp* copies per ng of total DNA (see Le Clec'h et al 2012 doi.org/10.1371/journal.ppat.1002844).

*Wolbachia* load varied significantly between females’ tissues (LRT = 35.06, P < 0.0001, **Figure S1**). *Wolbachia* was less abundant in the haemolymph than in the nerve chain and ovaries (LRT = 9.35, P = 0.002, LRT = 35.04, P < 0.0001, respectively). *Wolbachia* density was significantly lower in the nerve chain than in the ovaries (LRT = 15.57, P < 0.0001). On average, we found 1392 ± 206 bacteria per DNA ng in the haemolymph, 3781 ± 986 bacteria per DNA ng in the nerve chain and, 13897± 3548 bacteria per DNA ng in the ovaries. We therefore implemented a dilution procedure to standardize the concentration of *Wolbachia* in each tissue solution used to transfect the bacteria (see main text).


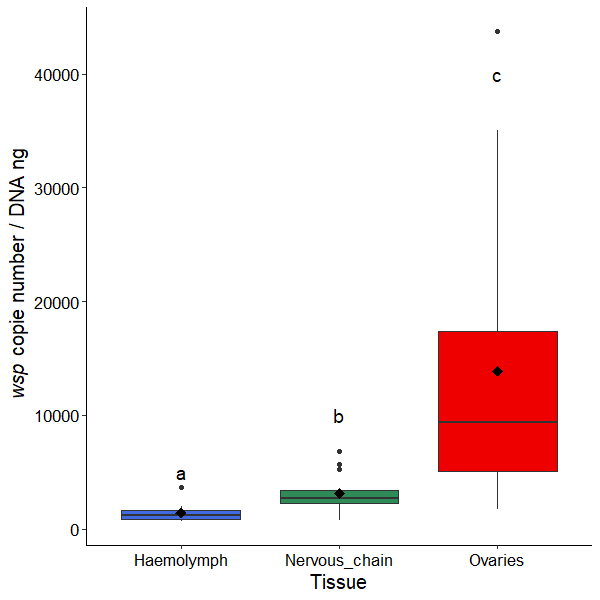


**Figure S1: wsp copy number per DNA ng in Armadillidium vulgare tissues**. Boxes above and below the medians (horizontal lines) show the first and third quartiles, respectively. Black diamonds represent the means. Levels not connected by the same letter are significantly different.

# Estimation of the proportion of live *Wolbachia* in filtered tissue solutions injected into recipient hosts.

To estimate the proportion of live *Wolbachia* in filtered tissue solutions injected into recipient individuals, we initially opted for a flow cytometry approach with double labelling of bacteria with propidium iodide (PI, whose fluorescence is recorded in the ECD channel) and Syto24 (whose fluorescence is recorded in the FITC channel). This protocol works well for bacteria pure cultures or water samples (Berney et al., 2007, doi: 10.1128/AEM.02750-06). In our case, filtered tissue solutions constitute complex samples, (e.i., including numerous cell debris, mitochondria, bacteria), so it was impossible to identify a clearly defined bacterial population from the classical FSC vs SSC plot. To circumvent this problem, we used the plot FSC vs Syto 24 to define a normalization gate encompassing the main populations common to all samples, infected by *Wolbachia* or not (example for nervous chain samples are displayed on Fig S2.A). Then, a population present in only all infected samples was defined as the *Wolbachia* one (Fig S2 B). Finally, IP staining was evaluated within the Wolbachia gate for each sample (Fig S2.C). Signal was very low, so that we didn’t consider counting of dead *Wolbachia* cells as being strong enough for statistical analyses, nevertheless it seemed that IP staining was not really different between infected samples and those which are not, thus suggesting that viability of the bacteria was very weakly compromised.


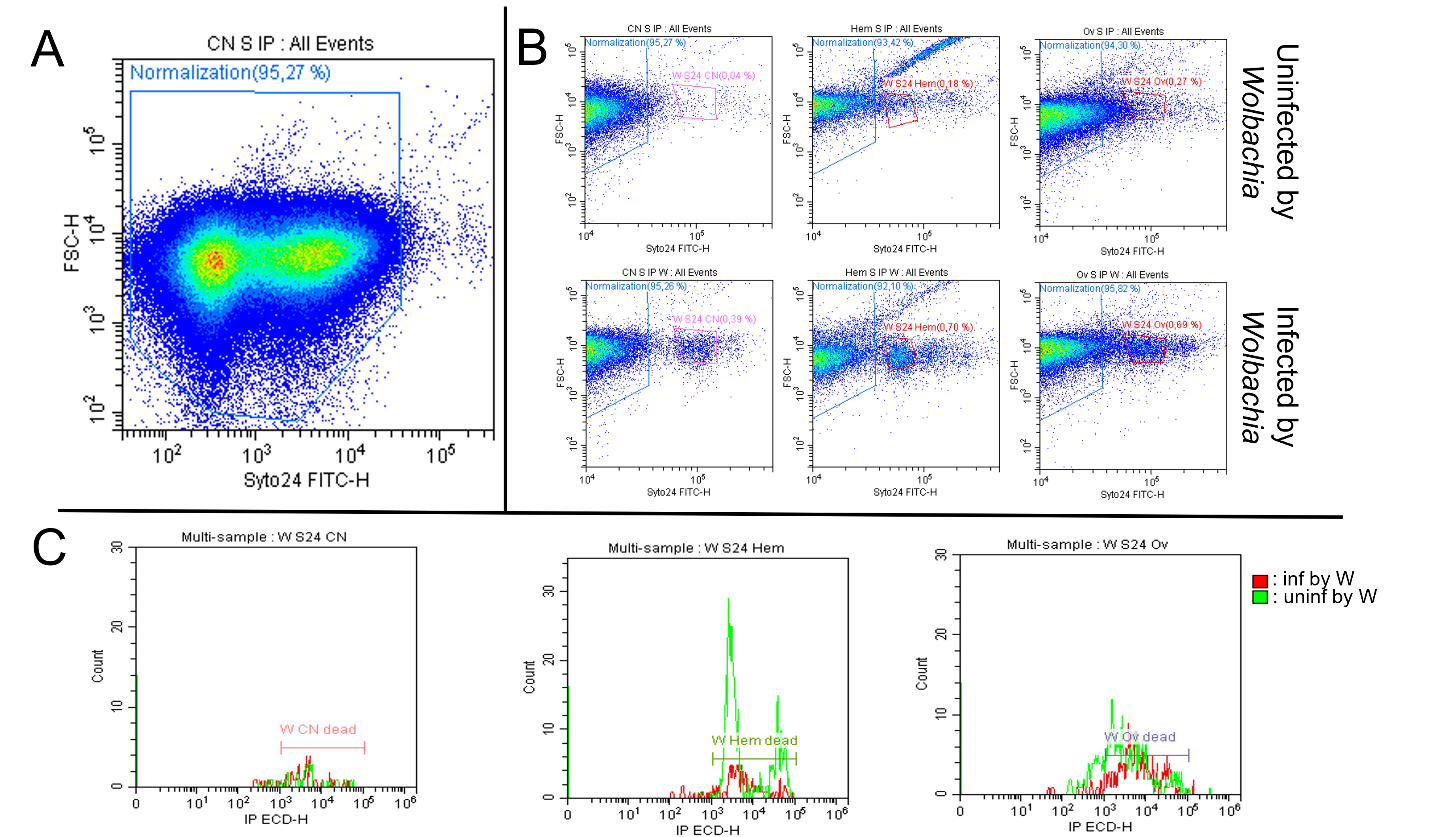


**Figure S2** **Flow cytometry analysis of Wolbachia-infected and non-infected filtered tissue solutions** (**A**) set of events measured in a sample (here a filtered nerve chain solution). This figure illustrates the gating of the main populations use to normalize acquisition for every sample (245000 events counting within the Normalization gate). (**B**) Identification of the gate containing Wolbachia. To do this, we compared the scatterplots between tissue filtrates produced from infected females versus tissue filtrates produced from uninfected females. This figure illustrates the fact that the number of events that appear to be associated with Wolbachia is very low (less than 1% of total events). (**C**) Quantification of fluorescent events due to Propidium Iodide staining in the Wolbachia gate. (CN, nervous chain; Hem, hemocytes; Ov, ovary; W, Wolbachia infected). This entire experiment was replicated three times without ever giving better results.

We used an alternative molecular approach to infer bacterial viability indirectly through RNA quantification (see Matsuda et al. 2007, doi:10.1128/AEM.01224-06). Specifically, we analyzed tissue-origin effects as a proxy for bacterial viability by comparing the ratio between the reverse transcriptase qPCR (RT-qPCR) cycle threshold (CT) values to DNA-based qPCR CT values, both performed on RNA and DNA extracted from the same tissue filtrates. To achieve this, we prepared filtered tissue solutions from five females, as presented in the main text. DNA and RNA were then extracted from 80µL of each solution. DNA and RNA extraction was performed using standard protocols (DNA extraction: Qiagen DNeasy 96 Blood & Tissue kit, RNA extraction: Macherey-Nagel Nucleozol). DNA-based qPCR was performed as described in the main text, and RT-qPCR was carried out using the Luna® Universal One-Step RT-qPCR Kit. Each sample was replicated (technical replicate and a total of four biological replicates were performed (i.e., four filtered ovary solutions, four filtered nerve chain solutions and one filtered hemolymph solution). This experiment highlighted that the proportion of live bacteria in the different filtered tissue solutions seems to be similar (LRT = 4.0279, p = 0.1335, Ratio ± 95%CI, CT RNA - CT DNA ratio: hemolymph = 0.815 ± 0.017, nervous chain = 0.875 ± 0.014, ovaries = 0.862 ± 0.037).

# (3) No significant interaction between the colonized recipient tissue and the *Wolbachia's* original tissue.

During the early infection period (between 20 et 60 days post-infection) there was no significant interaction between the colonized recipient tissue and the Wolbachia's original tissue (LRT = 6.957, p = 0.138)


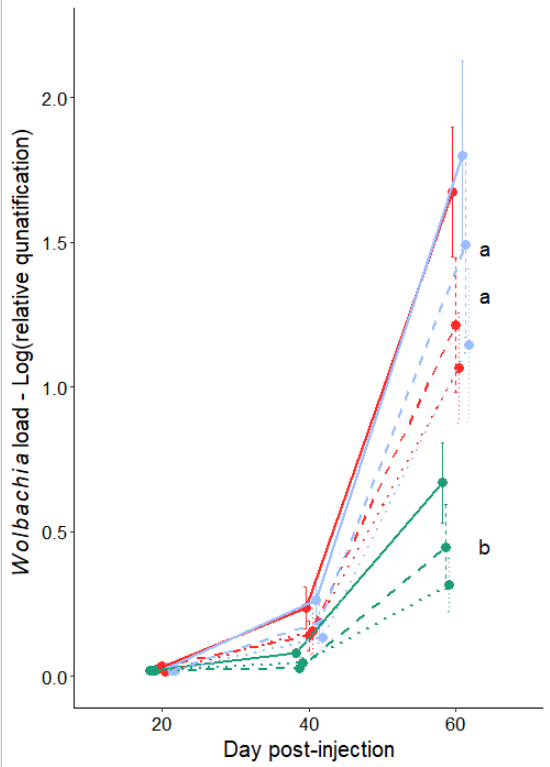


**Figure S3: Infection dynamics of Wolbachia (wVulC) in Armadillidium vulgare from day 0 to day 60 post-injection.** Colours represent the tissular origin of Wolbachia (red: ovaries, green: nerve chain, blue: haemolymphe) and line type the colonized tissue (recipient, solid = nerve chain, dotted: haemolymphe, dashed: ovaries)

# (4) Screening for variants in maternal lines

To determine whether the *Wolbachia* variant(s) validated by our filtration procedure (see above) arose during the focal individuals' lifetime or were maternally inherited, we searched for these variants in their maternal lineage. Specifically, we extracted DNA from the ovaries of sisters and maternal ancestors of focal individuals, going back six generations thanks to our broodstock collection stored at -20°C. The variants as well as the *Wolbachia* reference lineage were searched by amplification-refractory mutation system (ARMS, C. R. Newton et al., 1989). For this purpose, for each variant we designed a couple of primers targeting either the reference allele or the variant allele (see below). ARMS analyses were performed in a 25 µl reaction volume containing 0.5 µl genomic DNA, 5 µl 5X buffer (Promega), 0.5 µl dNTP, 1.25 µl forward and reverse primers and 0.125 µl GoTaq® polymerase (Promega). The volume was adjusted to 25 µl with double-distilled water. PCR regimen was as follows: initial denaturation at 95°C for 5 min, followed by 35 cycles for 30 s at 95°C, 30 s at 61°C, 30 s at 72°C, and then a final extension for 5 min at 72°C, and finally the PCR products were maintained at 4°C in the end. PCR products were separated on a 1.5% agarose gel (23min., 100V).

# Primers used for the ARMS PCR & Results.

**-Nucleotide sequence containing the SNP** (highlighted in yellow)

CTGGATTTCCATGGACCTTATTTACTCTACGCACT**C**GTCTCCTCTTTGGAGGAGCAGGTGGGCACACAGCTACCTTTGGTTTTTCAGTTTTGTCTTCTTTTTTACTCACTTTCTCCTGTTTCTGTGTTTCATTATTTTTGTGGTTTATAACAAATTTAGGTGAAGTGTCTACTAATTTTTCTACAGGTAAAAGAGCACGTTCTTGCTT

- **Primers used to target the reference sequence** (i.e., the *Wolbachia* lineage reported in all infected females)

Forward primer: TCCATGGACCTTATTTACTCTACGCACA**C**

Reverse primer: GCAAGAACGTGCTCTTTTACCT

- **Primers used to target the variant**

Forward primer : TAATAATAATCCATGGACCTTATTTACTCTACGCACA**T**

Reverse primer : GCAAGAACGTGCTCTTTTACCT

ARMS PCR results:


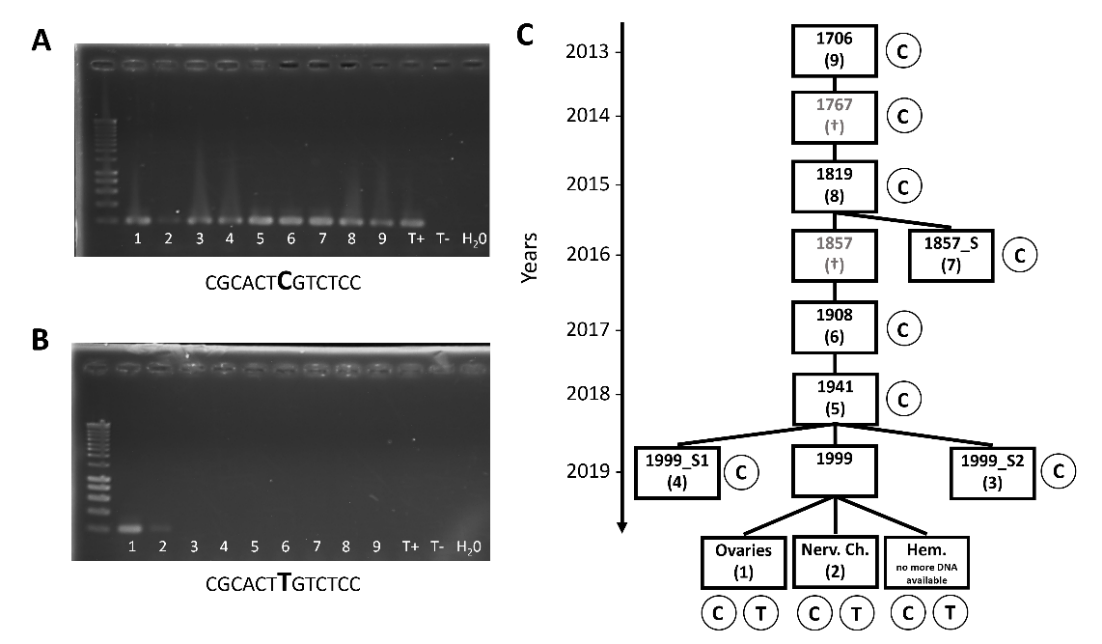


**Figure S4: ARMS PCR results and maternal genealogy of individual 1999.**
(**A**) Agarose gel (1.5%) showing PCR products obtained using primers targeting the Wolbachia reference sequence. (**B**) Agarose gel (1.5%) showing PCR products obtained using primers targeting the Wolbachia variant sequence. Lanes 1–9 correspond to individual females, whose identities are shown in (**C**). **T+**: Control female (line WXw) infected with Wolbachia but unrelated to individual 1999. **T−**: Control female (line WXa) uninfected by Wolbachia. **H₂O**: Negative control.
(**C**) Maternal genealogy of individual 1999, showing coinfection by two Wolbachia lineages as revealed by whole-genome resequencing on DNA from ovaries, nerve chain, and haemolymph (see table 1 in the main text). All the DNA extracted from the haemolymph of the 1999 female was used for whole-genome resequencing, which precluded validation of ARMS PCR in this sample. Two sisters of individual 1999 (1999_S1 and 1999_S2) were included in the analysis. If a direct maternal ancestor was unavailable (†: deceased before freezing), one of these sisters was used when possible. The circled letters represent the nucleotide observed at the variable position, with two letters indicating coinfection.

**Reference:**

Newton, C. R., Graham, A., Heptinstall, L. E., Powell, S. J., Summers, C., Kalsheker, N., Smith, J. C., & Markham, A. F. (1989). Analysis of any point mutation in DNA. The amplification refractory mutation system (ARMS). *Nucleic Acids Research*, *17*(7), 2503–2516. <https://doi.org/10.1093/nar/17.7.2503>

# **(5) IGV screenshot showing the alignment of reads realigned with bwa but without the pair-end option**


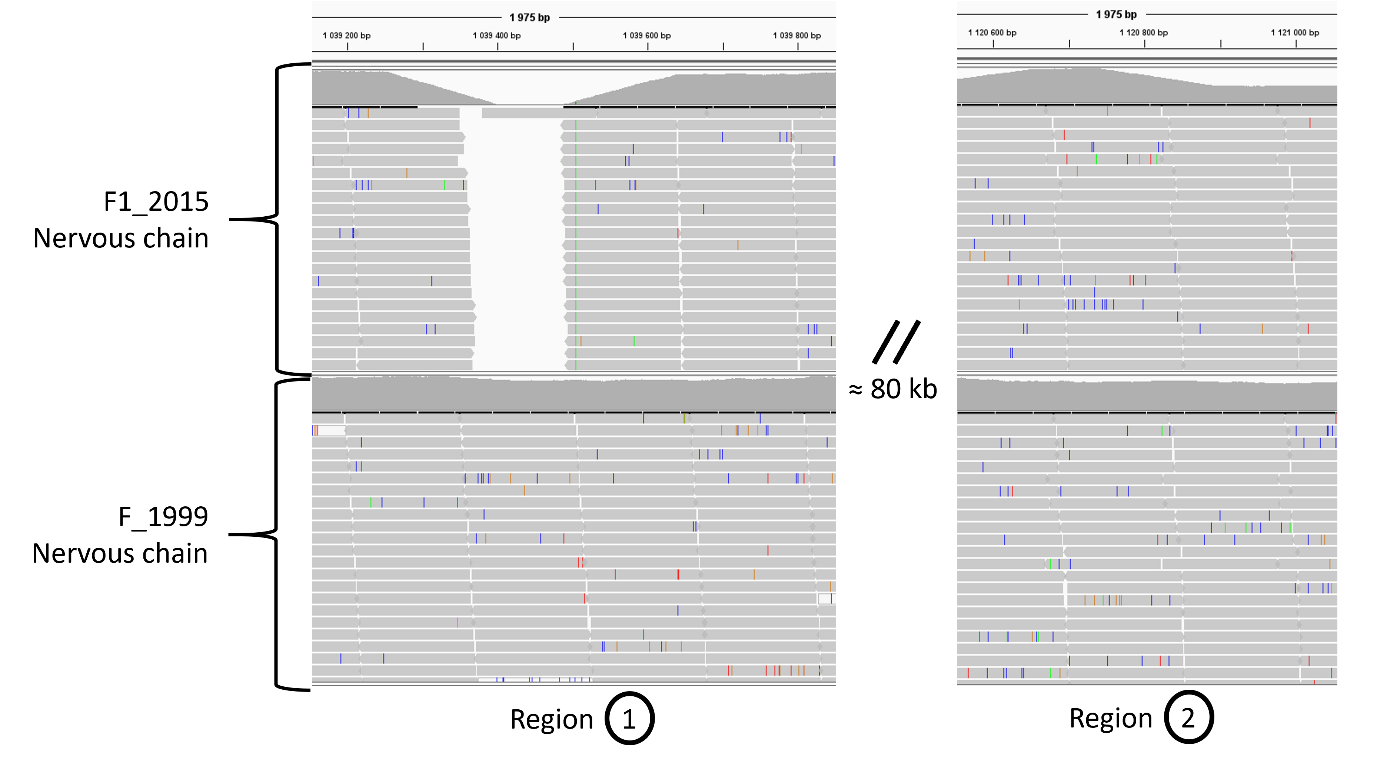


**Figure S5: IGV screenshot showing the alignment of reads from female F1_2015 and female F_1999 on region 1 and region 2 of the reference genome without the pair-end option.** Gene conversion event is suspected in the two sister females (F1_2015 and F2_2015). Each grey segment corresponds to a read.

# (6) Relationship between *Wolbachia* relative quantification in host’s tissues estimated by qPCR and using Illumina reads (Figure S6).

We aimed to determine whether the relative quantification of *Wolbachia* in different host tissues was consistent when measured by qPCR versus using Illumina sequencing reads obtained from the same samples. First, we quantified *Wolbachia* by qPCR as described in the Materials and Methods section of the manuscript. Then, we used the Illumina sequencing reads to estimate the relative *Wolbachia* load. To do this, we used samtools depth to calculate sequencing depth over the DNA regions of both the host and Wolbachia that are targeted by the qPCR primers. We then calculated the depth ratio (Wolbachia / host). Figure S5 shows a strong correlation between the two types of measurements (t = 9.74, p = 0.00019, Adjusted R-squared: 0.9399). This figure also shows that the bacterial densities in the different tissues of the F_1999 female fall within the range of bacterial densities measured in her two daughters, F1_2015 and F2_2015.


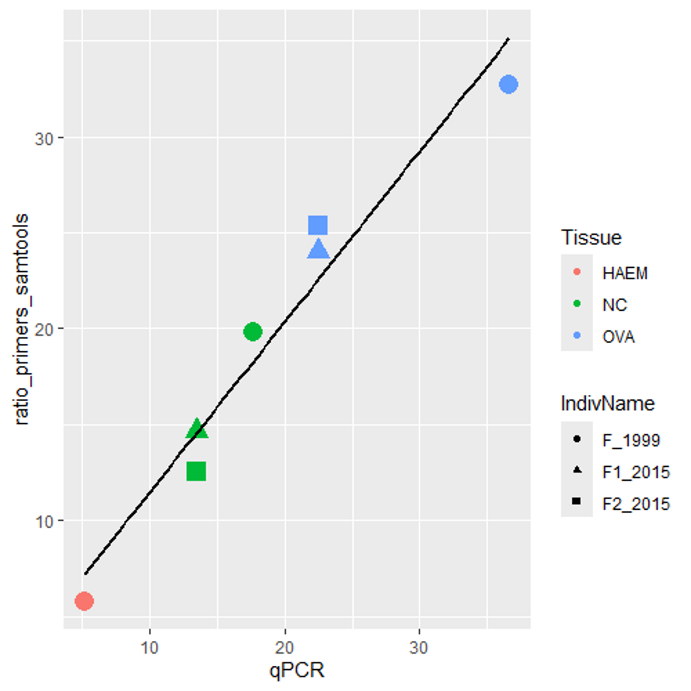


**Figure S6: Relationship between the relative quantification of Wolbachia estimated by qPCR or using sequencing depth.** HAEM: haemolymph, NC: nervous chain, OVA: ovaries
